# Suspecting Cardiac Amyloidosis in Congestive Heart Failure

**DOI:** 10.7759/cureus.11046

**Published:** 2020-10-19

**Authors:** Emad U Alatassi, Alaa Mohamed, Salim Habib, Iyiad Alabdul Razzak, Anas Mahmoud

**Affiliations:** 1 General Practice, Al Noor Specialist Hospital, Makkah, SAU; 2 Internal Medicine, California Institute of Behavioral Neurosciences & Psychology, Fairfield, USA; 3 Internal Medicine, Memorial Hermann-Texas Medical Center, Houston, USA; 4 Internal Medicine, University of Damascus, Damascus, SYR; 5 Internal Medicine, Alfaisal University-College of Medicine, Montgomery, USA; 6 Internal Medicine, Icahn School of Medicine at Mount Sinai, Queens General Hospital, New York, USA

**Keywords:** transthyretin amyloid cardiomyopathy, amyloidosis

## Abstract

Amyloidosis is a rare multisystem disease due to deposition of abnormal protein fragments, and cardiac amyloidosis is progressive and difficult to diagnose due to its subtle and non-specific symptoms unless the physician maintains a high degree of suspicion. This case report focuses on amyloid deposition in the heart of an 84-year-old woman who presented with symptoms of uncompensated heart failure.

## Introduction

Amyloidosis is a spectrum of diseases that used to go undiagnosed due to a lack of specific clinical diagnosis. It can be localized to a single organ or have systemic, multi-organ involvement. Each subtype of amyloidosis has its own prognosis and management plan. With the advancement in imaging techniques, amyloidosis is becoming increasingly identified and diagnosed much earlier [1]. A major prognostic factor is an early diagnosis, allowing earlier intervention limiting the disease progression.

## Case presentation

An 84-year-old African-American woman with a past medical history significant for congestive heart failure, hypertension, diabetes, and chronic kidney disease presented to the emergency department with shortness of breath and lower extremity swelling for over a year, which had worsened over the past week. She denied chest pain but admitted to having orthopnea and paroxysmal nocturnal dyspnea associated with productive cough with whitish frothy sputum.

**Figure 1:**
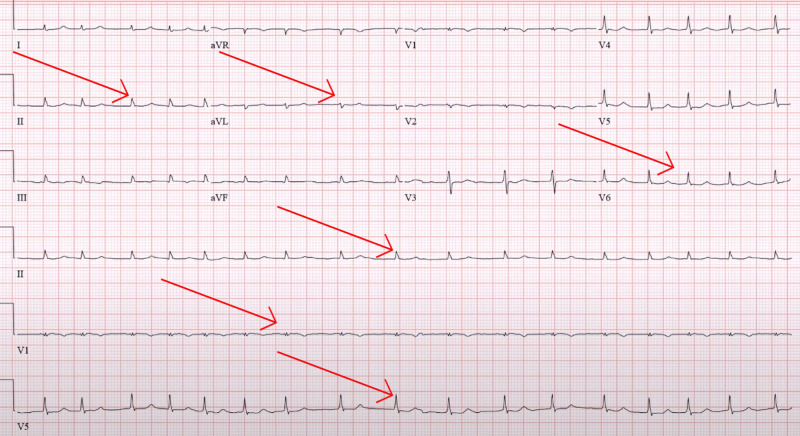
Arrows showing low voltage ECG. ECG: electrocardiogram

**Figure 2:**
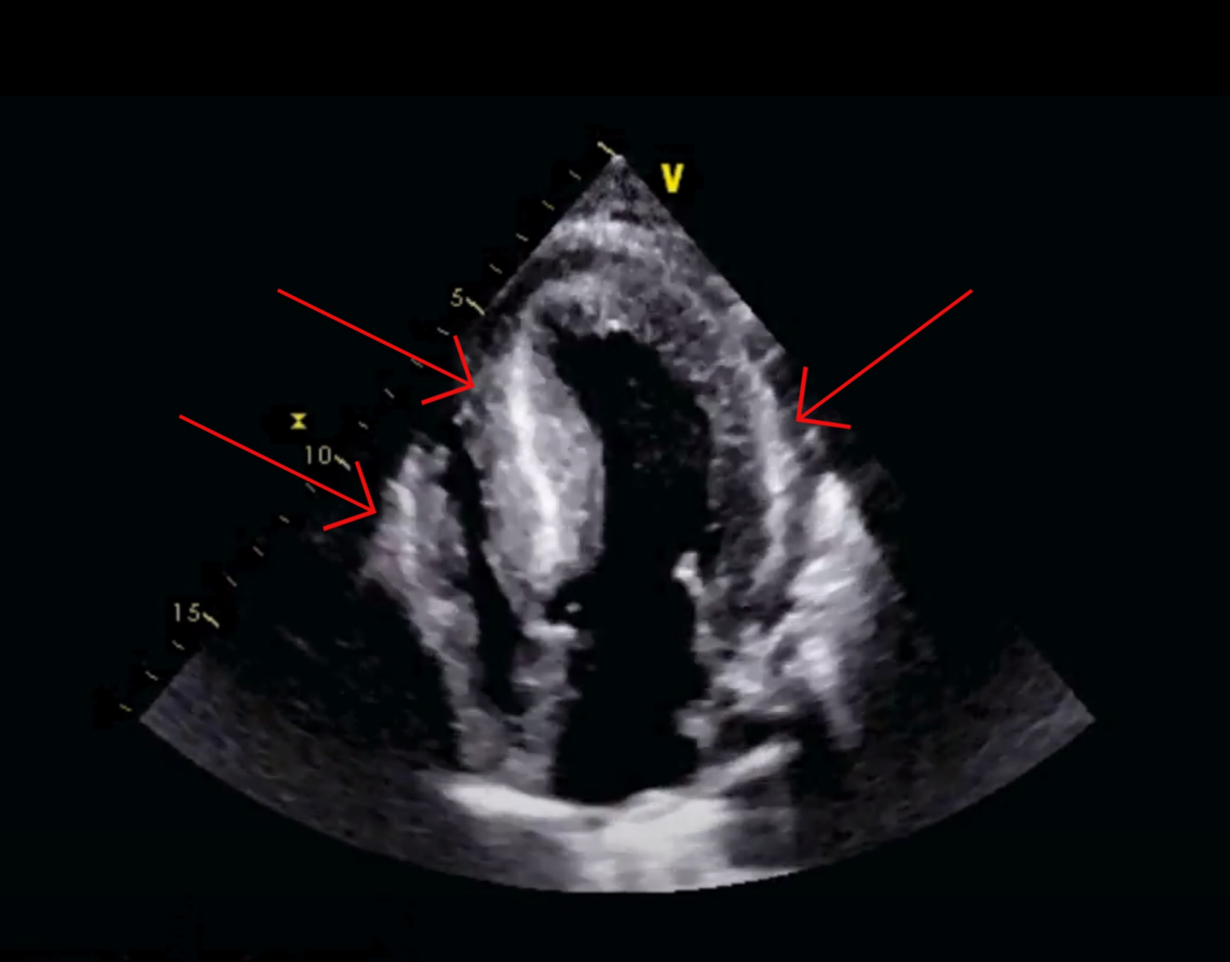
Echocardiogram showing thickened ventricular walls (arrows)

On examination, she appeared vitally stable but lethargic and could answer questions comfortably. We noted jugular venous distention along with hepatojugular reflux. On cardiac examination, we noted irregular rhythm and holosystolic murmur in the left sternal border. An exam of her lower extremities revealed edema (+2). An electrocardiogram (ECG) showed low voltage with controlled atrial fibrillation (Figure 1). Her bedside echocardiogram showed a reduced left ventricular ejection fraction of 25%, with concentric hypertrophy (Figure 2). Table 1 presents the results of her laboratory investigations.

**Table 1:** Blood work WBC: white blood cell; TSH: thyroid-stimulating hormone; NT-proBNP: N-terminal brain natriuretic peptide; BUN: blood urea nitrogen; K: potassium.

Analyte	Results (Reference Range)
Hemoglobin (g/dL)	11.2 (11-15)
WBC (cu/mm)	6400 (4000-12000)
TSH (μIU/mL)	3.4 (0.5-5.0)
Troponin T (ng/l)	26 (<30)
NT-proBNP (ng/l)	1130 (<125)
Creatinine (mg/dl)	5.5 (0.7-1.2)
BUN (mg/dl)	53 (7-30)
K (mmol/l)	5.2 (3.5-5.0)

She was admitted as a case of uncompensated congestive heart failure. The management plan focused on stabilizing the patient, treating her acute decompensation, and eventual referral to a specialized cardiology center for further investigation and optimization. We started her on diuretics, and the nephrologist started hemodialysis, given the patient's worsening renal function and volume overload. Her acute decompensation improved following dialysis, and the patient was discharged home with instructions to follow-up with her cardiologist and nephrologist. In her cardiology follow-up, the patient was diagnosed with suspected cardiac amyloidosis, but the patient refused further investigation.

## Discussion

Amyloidosis is the deposition of a pathologically misfolded precursor protein into a ß-pleated sheet form. The misfolded protein is insoluble and deposits in the extracellular matrix, distorting tissue architecture leading to organ dysfunction [1]. It can be systemic or localized to a specific organ like the heart, which is the focus of this patient's case.

Multiple types of amyloidosis are identified histochemically, but two main types comprise 95% of all cardiac amyloidosis. The first type is immunoglobulin light chain amyloidosis (AL), previously known as primary amyloidosis. AL has monoclonal light chains secondary to a plasma cell dyscrasia, presenting in patients as young as their thirties with a median age of 62. AL is usually a systemic disease with multi-organ involvement and is quite aggressive with rapid progression [2,3].

Transthyretin amyloidosis (ATTR) is the second major type of amyloidosis; ATTR is derived from transthyretin, produced by the liver affecting older male patients with a median age of 75 years and has a slower progression than AL. In general, ATTR carries a better prognosis than AL [4,5].

The clinical picture of amyloidosis varies depending on the organ predominantly involved, mostly kidneys, liver, and heart. The cardiac involvement usually presents as restrictive heart failure, systolic and diastolic dysfunction, conductive system disruption leading to arrhythmia and ischemia, pericardial effusion, ascites, lower limb edema, and hepatomegaly [6].

Cardiac amyloidosis is uncommon and usually diagnosed late due to its non-specific symptoms at initial presentation. However, early diagnosis is an important prognostic factor [7].

An ECG combined with an echocardiogram should raise the suspicion for cardiac amyloidosis, according to a series of ECG findings in 196 consecutive patients referred for cardiac amyloidosis biopsy [8]. In that study, the two main common noninvasive findings were low voltages on ECG and increased intraventricular septal thickness.

Echocardiogram findings in a patient with cardiac amyloidosis would be significant for left ventricular hypertrophy with a wall thickness of over 12 mm in the absence of systemic arterial hypertension. Other relevant findings consist of biatrial size increase with normal-sized ventricle, pericardial effusion, and, lastly, restrictive cardiomyopathy with diastolic dysfunction [9]. Cardiac magnetic resonance imaging (MRI) and technetium pyrophosphate (Tc-PYP) imaging are also used in establishing the diagnosis as MRI has a sensitivity and specificity of 85% to 90% and a positive predictive value of 90% in detecting cardiac amyloidosis [10]. Endomyocardial biopsy is the gold standard of diagnosis with 100% sensitivity for the collection of at least four samples [7]. Given that each type of amyloid progresses differently and is treated differently, it is important to detect and determine each type promptly.

Treatment for cardiac amyloidosis focuses on treating cardiac congestion by diuretics and is informed by the type of amyloidosis. Due to ALs plasma cell origin, AL can be targeted by different cytotoxic chemotherapies such as bortezomib, dexamethasone, and alkylating agents, followed by autologous stem cell transplant in eligible patients [11]. For patients with ATTR, tafamidis improved the quality of life and stabilized 97% of patients with wild type ATTR in a multicenter study [12]. Other drugs, such as diflunisal, doxycycline, and tauroursodeoxycholic acid showed effectiveness in other cases [8].

Organ transplants are another option for treatment. A liver transplant is a viable option given that a genetically normal donor liver will produce normal transthyretin. An orthotopic heart transplant is still somewhat controversial due to the recurrence of disease in transplanted hearts. Heart and liver transplant combined can be considered in ATTR patients if there are no other comorbidities [13]. Heart transplantation is palliative in AL but more successful in ATTR due to the absence of amyloid deposition. Heart transplantation's success is also due to isolated involvement of the heart in most cases, and late-onset and slow progression of cardiac involvement of ATTR compared to AL [13,14]. However, recurrence is still possible, as seen in other studies, requiring long-term follow-up [14].

In a recent study, 31 patients (both with AL and ATTR amyloidosis) underwent heart transplantation. In the post-transplantation period, there were no significant differences in rejection, malignancy, or mortality “between patients who underwent heart transplantation for amyloid cardiomyopathy and patients who underwent heart transplantation for all other indications” [15]. Barrett et al. note that “in carefully selected patients with cardiac amyloidosis, heart transplantation can be an effective therapeutic option with outcomes similar to those transplanted for other causes of heart failure” [15].

Age is one of the selection criteria for a heart transplant, and early reports indicated that with increased age, an increase in mortality ensues post-transplant, as seen in the Bourge et al. study [16]. The International Society for Heart and Lung Transplant Registry cited a significant decrease in survival in patients older than 65 years old [17]. However, a newer study of 519 patients showed that mortality and morbidity are similar in patients age 70 years or older compared to younger recipients. Therefore, advanced age alone should not be a contraindication and should not be an exclusion criterion [18].

## Conclusions

Cardiac amyloidosis remains a rare disease that should be seriously considered in any case presenting with non-specific symptoms that are usually suggestive of heart failure. Diagnosis can be difficult, and cardiac amyloidosis is often an undiagnosed cause of heart failure. ECG combined with echocardiography is an excellent noninvasive starter investigation followed by confirmatory cardiac MRI, Tc-PYP imaging, and endomyocardial biopsy for diagnosis and subtyping. Pharmacological and surgical treatment options are promising, but subtypes and early diagnosis dictate the prognosis.

## References

[1] Merlini G, Bellotti V (2003). Molecular mechanisms of amyloidosis. N Engl J Med.

[2] Gertz MA, Rajkumar SV (2002). Primary systemic amyloidosis. Curr Treat Options in Oncol.

[3] Dubrey SW, Cha K, Anderson J, Chamarthi B, Reisinger J, Skinner M, Falk RH (1998). The clinical features of immunoglobulin light-chain (AL) amyloidosis with heart involvement. QJM.

[4] Grogan M, Scott CG, Kyle RA (2016). Natural history of wild-type transthyretin cardiac amyloidosis and risk stratification using a novel staging system. J Am Coll Cardiol.

[5] Gillmore JD, Damy T, Fontana M (2018). A new staging system for cardiac transthyretin amyloidosis. Eur Heart J.

[6] Dungu JN, Anderson LJ, Whelan CJ, Hawkins PN (2012). Cardiac transthyretin amyloidosis. Heart J.

[7] Falk Falk, RH. RH. (2005). Diagnosis and management of the cardiac amyloidosis. Circulation.

[8] Rahman JE, Helou EF, Gelzer-Bell R (2004). Noninvasive diagnosis of biopsy-proven cardiac amyloidosis. J Am Coll Cardiol.

[9] Gertz MA, Benson MD, Dyck PJ (2015). Diagnosis, prognosis, and therapy of transthyretin amyloidosis. J Am Coll Cardiol.

[10] Ruberg FL, Appelbaum E, Davidoff R (2009). Diagnostic and prognostic utility of cardiovascular magnetic resonance imaging in light-chain cardiac amyloidosis. Am J Cardiol.

[11] Sperry BW, Ikram A, Hachamovitch R, Valent J, Vranian MN, Phelan D, Hanna M (2016). Efficacy of chemotherapy for light-chain amyloidosis in patients presenting with symptomatic heart failure. J Am Coll Cardiol.

[12] Maurer MS, Grogan DR, Judge DP (2015). Tafamidis in transthyretin amyloid cardiomyopathy: effects on transthyretin stabilization and clinical outcomes. Circ Heart Fail.

[13] Sousa M, Monohan G, Rajagopalan N, Grigorian A, Guglin M (2017 ). Heart transplantation in cardiac amyloidosis. Heart Fail Rev.

[14] Fuchs U, Zittermann A, Suhr O, Holmgren G, Tenderich G, Minami K, Koerfer R (2005). Heart transplantation in a 68-year-old patient with senile systemic amyloidosis. Am J Transplant.

[15] Barrett CD, Alexander KM, Zhao H (2020). Outcomes in patients with cardiac amyloidosis undergoing heart transplantation. JAAC Heart Fail..

[16] Bourge RC, Naftel DC, Costanzo-Nordin MR (1993). Pretransplantation risk factors for death after heart transplantation: a multi-institutional study. The Transplant Cardiologists Research Database Group. J Heart Lung Transplant.

[17] Khush KK, Cherikh WS, Chambers DC (2019). The international thoracic organ transplant registry of the international society for heart and lung transplantation: thirty-sixth adult heart transplantation report.

[18] Daneshvar D, Czer LS, Phan A (2011). Heart transplantation in patients aged 70 years and older: a two-decade experience. Transplant Proc.

